# Breaking the Vicious Cycle? A Systematic Review of Interventions Targeting Both Falls and Fear of Falling in Older Adults

**DOI:** 10.3390/geriatrics11030072

**Published:** 2026-06-16

**Authors:** Asiye Tuba Ozdogar, Pervin Yesiloglu, Yuval Levitan Marcus, Alon Kalron

**Affiliations:** 1 Department of Physiotherapy, Faculty of Health Sciences, Van Yüzüncü Yıl University, 65080 Van, Turkey; tuba.ozdogar@yahoo.com (A.T.O.); pyesiloglu1@gmail.com (P.Y.); 2 Department of Physical Therapy, School of Health Professions, Gray Faculty of Medical and Health Sciences, Tel-Aviv University, Tel-Aviv 6997801, Israel; yuvallevitan@gmail.com; 3 Brain and Nervous System Health Division, Sheba Medical Center, Ramat-Gan 52621, Israel; 4 Multiple Sclerosis Center, Sheba Medical Center, Ramat-Gan 52621, Israel; 5 Sagol School of Neuroscience, Tel-Aviv University, Tel-Aviv 6997801, Israel

**Keywords:** falls, fear of falling, older adults, systematic review, balance interventions

## Abstract

Background: Falls and fall-related injuries are common in older adults and are frequently accompanied by fear of falling (FoF), which may lead to activity avoidance and functional decline. Because many interventions target falls or FoF in isolation, we conducted a systematic review and meta-analysis of randomized controlled trials (RCTs) to identify, describe, and evaluate interventions reporting both falls and FoF outcomes in older adults. Methods: This systematic review and meta-analysis were registered in PROSPERO (CRD420251113137) and conducted in accordance with PRISMA guidelines. PubMed, Embase, and Web of Science were searched from inception to 4 November 2025. Eligible studies were English-language RCTs that included adults aged ≥60 years, evaluated nonpharmacological interventions, and reported both FoF and falls. Methodological quality was assessed using the PEDro scale. Random-effects meta-analyses were performed for FoF (Hedges g), and Bayesian random-effects binomial meta-analyses were conducted for falls. Results: Ten RCTs published between 1998 and 2018 (sample sizes per trial: *n* = 27–540) were included. Interventions included cognitive–behavioral therapy-based programs, Tai Chi, physiotherapist-led strength and balance training, computerized visual feedback, and video-guided home exercise. PEDro scores ranged from 6 to 9 (mean, 7.7). Pooled analyses showed no significant effect on FoF at the end of intervention (g = −0.20, 95% CI −1.45 to 1.05; *p* = 0.68; high heterogeneity) or at follow-up (g = −0.14, 95% CI −0.60 to 0.33; *p* = 0.50). For falls, postintervention evidence favored the null (BF_10_ = 0.16; pooled estimate −0.01, 95% credible interval [CrI] −0.30 to 0.14). Follow-up results were inconclusive (BF_10_ = 2.07; pooled CrI −0.56 to 0.00), with substantial uncertainty. Conclusions: Across RCTs that measured both outcomes, interventions did not consistently improve both FoF and falls outcomes. These findings may suggest a partial dissociation between psychological and physical fall-related outcomes, highlighting the need for integrated, adequately powered trials that utilize standardized measures and longer follow-up periods.

## 1. Introduction

Fall-related injuries are common among older adults and represent a major cause of fractures, pain, and functional decline, often leading to disability, loss of independence, reduced quality of life, and even premature death [1]. According to the World Health Organization (WHO) and national health reports, approximately 28–35% of adults aged 65 years and older experience at least one fall each year, with the proportion rising to 32–42% among those aged 70 years and older [2,3,4]. The financial burden associated with falls in older populations is considerable and continues to increase globally [5]. Beyond the economic impact, the personal and societal consequences of fall-related injuries include reduced autonomy, caregiver strain, and increased healthcare utilization [6]. This highlights the significance of this issue as a global public health concern.

Beyond the physical consequences of falls, many older adults develop a persistent fear of falling (FoF), also called concern about falling, which can profoundly affect well-being and daily functioning [7]. FoF is highly prevalent among older adults, reported in up to half of community-dwelling individuals, and may persist long after a fall or even occur in those who have never experienced one [8]. When sustained, FoF becomes a significant health problem, leading to activity avoidance, physical deconditioning, reduced participation in daily and social activities, and diminished quality of life [7,8].

Although FoF and falls are closely linked, research indicates that they do not always co-occur. Many older adults report FoF despite no recent or previous fall, whereas others who have fallen do not develop such fear [9]. The exact nature of this association remains unclear. As highlighted by previous research [9,10], multiple mechanisms may underlie the relationship, including avoidance and deconditioning, anxiety-related effects on balance control, or realistic appraisal of one’s own physical limitations. This complexity suggests that FoF should be considered both a potential consequence and an independent contributor to fall risk, warranting targeted attention in both prevention and rehabilitation strategies [11].

Various intervention strategies have been developed to mitigate fall risk in older adults, including exercise-based programs, multifactorial risk assessments, home hazard modifications, and educational approaches [12]. Separately, psychological interventions such as cognitive–behavioral therapy and self-efficacy training have shown promise in reducing FoF [13]. However, despite the strong bidirectional link between these phenomena, most research and clinical programs have focused on either falls or FoF in isolation. Evidence on interventions targeting both falls and FoF remains limited and heterogeneous, with substantial variation in intervention design, participant populations, outcome measures, and disciplinary approaches, making direct comparison and synthesis difficult.

A comprehensive synthesis of interventions addressing both falls and FoF is needed to guide integrated prevention and rehabilitation strategies. Understanding which approaches are most effective in simultaneously improving physical and psychological outcomes can inform the design of multifaceted, person-centered programs for older adults.

The aim of this systematic review and meta-analysis is therefore to identify, describe, and evaluate interventions that reported both falls and FoF outcomes among older adults. By summarizing the existing evidence, this review highlights characteristics of interventions that reported both falls and FoF outcomes and identifies gaps to inform future research and clinical practice.

## 2. Methods

### 2.1. Study Design

The review was registered in PROSPERO (registration number CRD 420251113137) and followed the Preferred Reporting Items for Systematic Reviews and Meta-Analyses (PRISMA) guidelines [14]. In accordance with the Population, Intervention, Comparison, Outcomes, and Study (PICOS) guidelines, we formulated the research question and constituted the following study inclusion and exclusion criteria:

### 2.2. Participants

Studies must involve participants aged 60 years or older.

### 2.3. Intervention

This systematic review aimed to synthesize evidence from randomized controlled trials evaluating nonpharmacological interventions that reported both falls and FoF outcomes in older adults. Eligible interventions included exercise-based, balance-training, cognitive–behavioral, educational, Tai Chi, and multifactorial rehabilitation approaches designed to address physical and/or psychological factors related to fall risk.

### 2.4. Comparison

Comparison groups included usual care, minimal educational materials (e.g., brochures or written information), social contact interventions, wait-list controls, home safety assessments without active training, or no-intervention control conditions. Studies comparing one eligible nonpharmacological intervention with another were also considered.

### 2.5. Outcomes

The primary outcome measures were FoF and fall incidence. Only studies where the present mean and standard deviation were included for meta-analysis.

### 2.6. Eligible Study Designs

The present review included randomized controlled trials conducted in any setting where interventions were applied to older adults (e.g., community-dwelling, outpatient clinics, rehabilitation centers, or nursing homes). Studies must involve participants aged 60 years or older. Only studies published in English were taken into consideration. Trials involving non-human participants or populations with specific neurological conditions (e.g., Parkinson’s disease and stroke) that primarily affect balance were excluded unless the primary aim was general fall prevention in older adults.

### 2.7. Search Strategy

All authors (A.T.O., A.K., Y.L., and P.Y.) thoroughly searched the MEDLINE (PubMed), Embase, Scopus, and Web of Science databases for relevant studies from inception through 4 November 2025, based on the predefined inclusion and exclusion criteria. The following keywords were used individually and in combination: (“older adults” OR “elderly” OR “aged”) AND (falls OR fall OR falling) AND (fear of falling) AND (“postural balance” OR balance OR “postural control” OR “dynamic balance” OR “postural stability” OR “postural equilibrium” OR “postural instability” OR “dynamic stability” OR “dynamic control”) AND (“intervention” OR “prevention” OR “rehabilitation” OR “treatment” OR “therapy”). Search was restricted to human studies published in English. Reference lists were also manually searched for all primary and randomized controlled trial articles. Although no medical librarian or information specialist was formally involved, the search strategy was collaboratively developed and refined by all authors through iterative testing across databases to maximize sensitivity and relevance.

### 2.8. Study Selection

Titles and abstracts were screened for eligibility based on our inclusion/exclusion criteria, following the PRISMA 2020 protocol [14]. Duplicate search results were removed, and afterward, the full texts of the remaining articles were thoroughly reviewed.

### 2.9. Data Extraction

Each of the four researchers independently extracted data and screened each record for eligibility. The extracted data from the articles were entered into a predefined database. The data included the authors’ names, the study title, year of publication, outcome, baseline, end of intervention, follow-up assessment time, and statistical significance. Study authors were not contacted for additional information.

### 2.10. Quality Assessment

Risk of bias was assessed using the Physiotherapy Evidence Database (PEDro) scale. Those scoring at least 6 (out of 11) were considered high-quality research. Data were independently assessed by at least two people with a process to resolve differences. If the necessary information was unclear or unavailable in the study publications/reports, additional information was requested from the study researchers.

### 2.11. Meta-Analysis

In this meta-analysis, random-effects models were used to estimate the pooled effects for both continuous and binary outcomes, consistent with recommendations for synthesizing heterogeneous clinical studies [15]. For the FoF outcomes, which were reported as continuous measures, a classical random-effects model was applied. Because FoF was assessed using different validated instruments across studies, pooled continuous outcomes were synthesized using standardized mean differences (Hedges g), enabling comparison across scales with different score ranges and distributions. This model assumes that the included studies represent a random sample from a broader distribution of possible studies, allowing the true effect size to vary across studies. Heterogeneity was assessed using the Q statistic and quantified with τ and τ^2^ estimates. The expected value of Q equals the degrees of freedom under the assumption of a common effect size, and significant deviations from this expectation indicate between-study heterogeneity. Prediction intervals were also calculated to describe the range in which true effects of similar future studies might fall.

Studies were included in the meta-analysis when sufficient quantitative outcome data were reported to permit calculation of pooled effect estimates at comparable time points. For falls outcomes, which were reported as counts of fallers and non-fallers (binary data), a Bayesian random-effects binomial meta-analysis was conducted using Bayes Factors to quantify the strength of the evidence [16]. A Bayesian approach was selected because of the limited number of eligible studies and sparse binary outcome data, conditions under which Bayesian estimation may provide more stable inference and direct quantification of uncertainty. The Bayesian framework produces pooled effect estimates (e.g., risk ratios) while quantifying the strength of evidence using Bayes Factors (BF_10_). Heterogeneity in the Bayesian framework was evaluated using τ and τ^2^, with Bayes Factors summarizing the evidence for the presence or absence of between-study variation. Prediction intervals (PI) were computed to illustrate the expected dispersion of true effects across new populations.

Across both analytic approaches, forest plots were generated to visualize individual study effects and pooled estimates. Relative study weights were calculated based on model variance components; studies with more precise estimates contributed a proportionally greater weight to the overall pooled effect. All analyses were performed using the JASP statistical software (version 0.19.0) Formal assessment of publication bias was not conducted because the number of included studies per meta-analysis was below the recommended threshold for interpreting funnel plot asymmetry or related statistical tests.

## 3. Results

### 3.1. Selection of Studies

**Figure 1:**
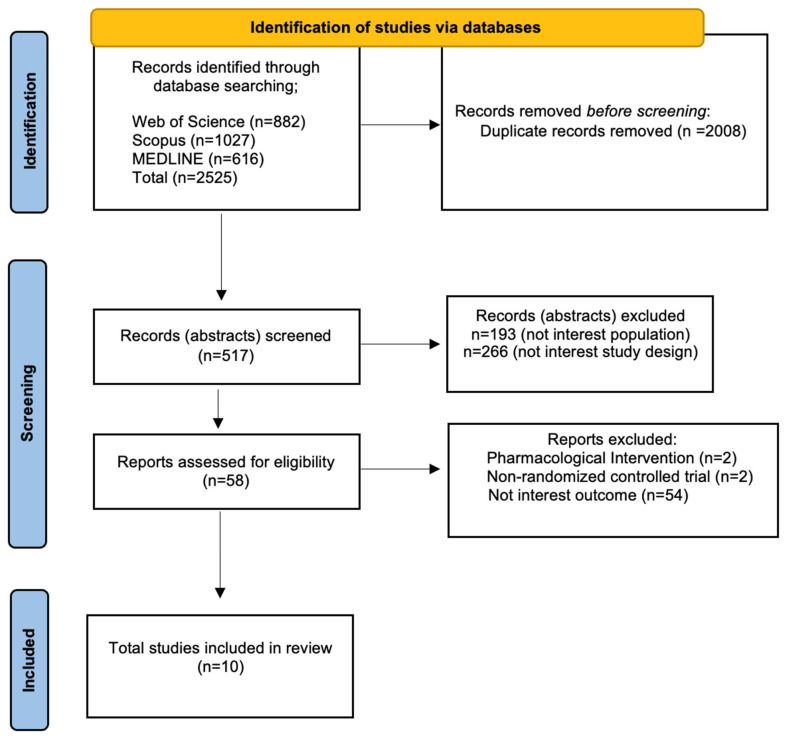
Flowchart of study selection.

This PRISMA flow diagram illustrates the process of selecting studies for the review. It begins by identifying 2525 records across three databases (Web of Science: 882; Scopus: 1027; and MEDLINE: 616), screening out 2008 duplicates, and further excluding 459 records during the abstract screening phase. Of the 68 reports assessed for eligibility, 58 were excluded based on the specific criteria, resulting in 10 studies in the final review. The flowchart of the systematic review is presented in Figure 1.

### 3.2. Characteristics of Included Studies

The included studies were conducted between 1998 and 2018 across several countries, including the Netherlands, Taiwan, the United States, Australia, and New Zealand. All studies employed an RCT design, with sample sizes ranging from small pilot groups of 27 participants to large community-based cohorts of up to 540 individuals and an average age of 78.46 years. Most trials recruited older adults with a history of falls or an elevated fall risk, with a mean age typically above 70 years. Follow-up durations varied considerably across studies, from immediate post-intervention assessments to 24-month follow-up evaluations.

Control conditions differed across trials and included usual care, educational brochures, waitlist control, home safety assessments without active training, or no-treatment controls. Intervention duration, frequency, and delivery format varied substantially across studies, ranging from short-term multi-session interventions to year-long community-based exercise programs (Table 1). Delivery formats included group-based sessions, structured home programs, video-guided exercise, Tai Chi classes, and cognitive–behavioral therapy (CBT) sessions.

FoF outcomes were measured using validated scales, such as the Falls Efficacy Scale (FES), Falls Efficacy Scale—International (FES-I), the Activities-specific Balance Confidence (ABC) Scale, and single-item FoF questions.

Secondary outcomes included fall incidence, fall counts, and faller proportions, which were collected through falls diaries, monthly monitoring, or retrospective reporting. Some interventions incorporated behavioral components to reduce fear, while others primarily emphasized physical training or balance enhancement. The varied follow-up durations, intervention intensities, and FoF measurement tools contributed to methodological heterogeneity across the included trials. A detailed summary of each study’s characteristics is provided in Table 1.

### 3.3. Interventions

The included studies examined a range of interventions targeting fear of falling (FoF) and fall risk, including cognitive–behavioral therapy (CBT) programs [17,18,19,20,21], Tai Chi–based movement interventions [18,25], physiotherapist-led strength and balance training [20,22,23], video-based guided home exercise programs [24], and computerized visual feedback balance training [26]. Although intervention formats varied, most combined physical exercise with cognitive–behavioral strategies and educational components. CBT-based interventions focused on modifying maladaptive beliefs, reducing activity avoidance, and enhancing self-efficacy related to falling [17,18,19,20,21], while exercise-based programs primarily targeted balance, strength, gait, and functional mobility [22,23,24,25,26]. Several studies have integrated both approaches within multi-component interventions delivered individually or in group settings, either at home or under supervision [18,20,21]. Intervention duration ranged from short-term intensive programs to long-term training protocols lasting up to two years [22,23].

### 3.4. Quality Assessment of Included Studies

The methodological quality of the included studies was assessed using the PEDro scale, which evaluates internal validity and statistical reporting across 11 criteria. As summarized in Table 2, PEDro scores across the ten included studies ranged from 6 to 9, indicating overall moderate to high methodological quality, with a mean score of approximately 7.7. Several studies achieved scores of 8 or higher, including Dorresteijn (2016) [17], Huang (2011) [18], Wetherell (2018) [20], Zijlstra (2009) [21], Barnett (2003) [23], and Logghe (2009) [25].

Most studies demonstrated strengths in random allocation, baseline comparability, blinded outcome assessment, between-group statistical analyses, and reporting of point estimates and variability, supporting internal validity. Common limitations included a lack of blinding of participants and therapists across all studies, a frequent constraint in physiotherapy and behavioral interventions, which may increase performance bias. In addition, inadequate follow-up or the absence of intention-to-treat analysis was noted in several studies, including those by Dorresteijn (2016) [17], Wetherell (2018) [20], Haines (2009) [24], and Sihvonen (2004) [26]. Overall, despite these limitations, the generally moderate-to-high PEDro scores support the methodological soundness of the included evidence.

### 3.5. Results of the Studies

#### 3.5.1. Fear of Falling

Across studies, several interventions demonstrated within-group improvements in FoF, but consistent between-group differences were uncommon. Significant within-group reductions were reported in CBT-based interventions, including Dorresteijn et al. (2016) [17], Tennstedt et al. (1998) [19], and Wetherell et al. (2018) [20], while Huang et al. (2011) reported improvements in both CBT and CBT + Tai Chi arms without statistically significant between-group differences [18]. Campbell et al. (1999) observed divergent trends favoring the intervention group [22], whereas Haines et al. (2009) [24] and Logghe et al. (2009) [25] reported minimal or no changes. Studies using single-item FoF measures also showed reductions within intervention groups, but adjusted between-group effects were generally non-significant [21,23,26] (Table 3).

**Figure 2:**
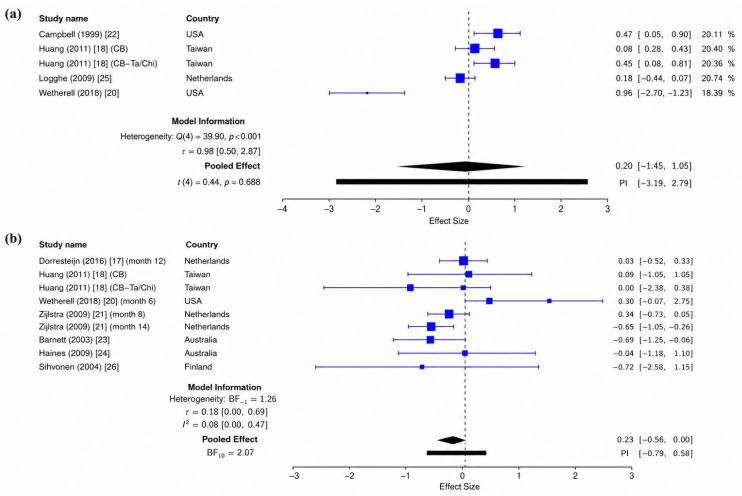
Meta-analysis investigating the effect of non-pharmacological interventions on FoF at the end of the intervention period (**a**) [18,20,22,25] and on FoF at the follow-up period (**b**) [17,18,20,21,23,24,26].

Post-intervention FoF outcomes from five studies were included in the meta-analysis. Individual effect sizes ranged from small negative to moderate positive values, with several confidence intervals crossing the line of no effect. Small to moderate effects favoring the intervention were observed in Huang et al. (2011; CBT and CBT + Tai Chi) [18] and Logghe et al. (2009; Tai Chi) [25]. In contrast, Campbell et al. (1999; home-based strength and balance training) [22] and Wetherell et al. (2018; integrated exposure therapy and exercise) [20] showed inconclusive effects. The random-effects model indicated no statistically significant pooled effect at the end of intervention (g = −0.20, 95% CI [−1.45, 1.05], *p* = 0.683), with substantial heterogeneity (Q(4) = 39.90, *p* < 0.001; τ = 0.98). The wide prediction interval (−3.19 to 2.79) suggested considerable variability in potential effects across settings (Figure 2a).

Five studies (seven assessments) reported follow-up FoF outcomes. Minor improvements favoring the intervention were observed in Dorresteijn et al. (2016) [17], whereas other studies reported wide confidence intervals and mixed or null effects [18,20,24,25]. The pooled follow-up effect was not statistically significant (g = −0.14, 95% CI [−0.60, 0.33], *p* = 0.499), with significant heterogeneity (Q(6) = 33.82, *p* < 0.001; τ = 0.46) and a broad prediction interval (−1.36 to 1.09), indicating substantial uncertainty regarding long-term intervention effects (Figure 2b).

#### 3.5.2. Falls

Across studies, fall outcomes demonstrated substantial variability, with most interventions failing to produce statistically significant reductions compared with control conditions. Several trials reported numerical reductions in fallers within intervention groups, including Dorresteijn et al. (2016) [17], Barnett et al. (2003) [23], and Huang et al. (2011; CBT + Tai Chi) [18], but between-group differences were generally non-significant. No meaningful effects were observed in Tennstedt et al. (1998) [19], Wetherell et al. (2018) [20], Zijlstra et al. (2009) [21], Campbell et al. (1999) [22], Logghe et al. (2009) [25], or Haines et al. (2009) [24]. Sihvonen et al. (2004) reported a statistically significant risk ratio favoring the intervention; however, this finding was based on a very small sample (*n* = 27) and should be interpreted with caution [26] (Table 3).

**Figure 3:**
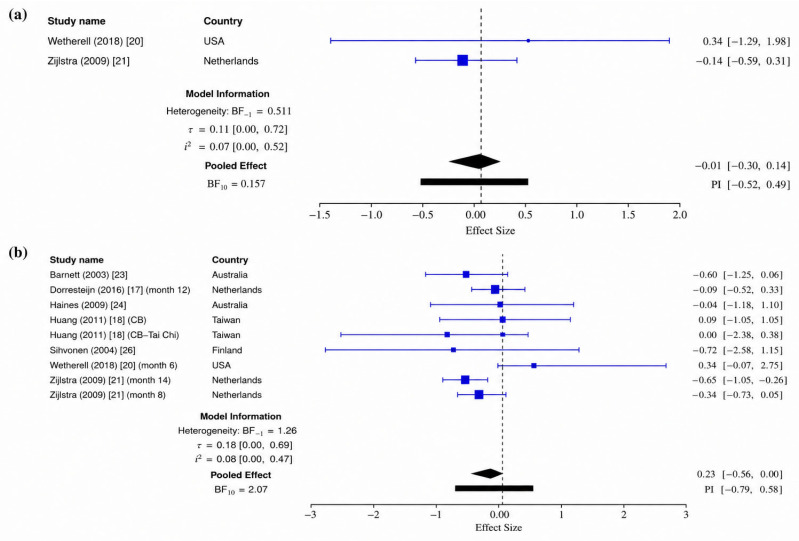
Meta-analysis investigating the effect of non-pharmacological interventions on falls at the end of the intervention period (**a**) [20,21] and on falls at the follow-up period (**b**) [17,18,20,21,23,24,26].

Two studies reported post-intervention fall outcomes suitable for meta-analysis [20,21]. Individual effect sizes were small and imprecise, with confidence intervals crossing the line of no effect. The Bayesian random-effects model provided no evidence for a pooled intervention effect (BF_10_ = 0.157), with a pooled estimate close to zero (−0.01; 95% credible interval: −0.30 to 0.14) and a wide prediction interval (−0.52 to 0.49). Between-study heterogeneity was minimal (τ = 0.11), although uncertainty remained high due to limited data (Figure 3a).

Seven studies (nine assessments) reported follow-up fall outcomes and were included in a second meta-analysis [17,18,21,23,24,25,26]. Effect estimates varied, with most studies showing effects close to zero and wide credible intervals. The Bayesian random-effects model yielded no strong evidence for a pooled effect (BF_10_ = 2.07), and the prediction interval (−0.79 to 0.58) indicated that future effects could plausibly favor either group. Heterogeneity was modest (τ = 0.18), indicating some variability across trials but no consistent benefit from the intervention (Figure 3b).

## 4. Discussion

A notable and clinically relevant finding of this systematic review and meta-analysis is that none of the interventions achieved simultaneous improvements in both FoF and actual fall incidence. Across the ten included trials, reductions in FoF were relatively common; five studies reported significant improvements, whereas only two demonstrated meaningful decreases in falls, and these effects were inconsistent over time and across study designs. Collectively, the included studies may suggest partial dissociation between psychological and physical fall-related outcomes; interventions that effectively reduce psychological fear do not necessarily minimize physical fall events, and vice versa. Accordingly, FoF and fall risk may not respond uniformly to the same interventions, underscoring the importance of assessing and addressing each domain directly rather than assuming that improvement in one will automatically translate to improvement in the other. Nevertheless, identifying an intervention capable of improving both FoF and falls remains a critically important goal, as a truly dual-effective approach could have substantial implications for clinical practice, prevention strategies, and overall functional independence in older adults.

The dissociation we observed aligns with emerging theoretical perspectives, such as the Falls–Fear–Avoidance Triad of Causation [10], which provides a relevant framework for understanding why interventions seldom influence both outcomes simultaneously. The Falls–Fear–Avoidance Triad of Causation proposes that falls, FoF, and activity avoidance interact dynamically and multifactorially rather than through a simple linear pathway, as described in the traditional fear-avoidance model of falling [27]. Depending on an individual’s physical capacity, psychological resilience, and ability to interpret internal bodily signals, FoF may trigger protective behaviors that reduce risk or maladaptive avoidance that contributes to deconditioning and increased likelihood of falls. Importantly, FoF may not always represent a maladaptive response. In some individuals, heightened caution may reflect appropriate risk awareness and protective behavioral adaptation, whereas in others, excessive fear may contribute to activity restriction and deconditioning. Differences in baseline psychological status, recurrent fall history, and physical capacity may therefore substantially influence intervention responsiveness and interpretation of FoF outcomes. Conversely, low or absent FoF, particularly in individuals with impaired balance, may remove essential caution and increase fall vulnerability. Applied to older adults, this framework highlights that FoF and falls are not only distinct constructs but may also interact in contradictory ways, depending on behavioral, cognitive, and physical mechanisms. Such complexity likely contributes to the varied response patterns reported in the literature and reinforces the need for multimodal strategies that address both the psychological and physical aspects of fall risk.

Notably, evidence from other neurological populations reveals a similar pattern of dissociation between improvements in FoF and reductions in fall incidence. In Parkinson’s disease, the RCT by Canning et al. (2015) showed that a minimally supervised exercise program significantly improved FoF and physical performance but did not reduce falls overall; fall reduction occurred only in participants with milder disease [28]. In stroke survivors, Batchelor et al. (2012) found no reduction in fall rate, proportion of fallers, or injurious falls, despite within-group improvement in fall-related efficacy, indicating that psychological gains alone did not translate into fewer falls [29]. In multiple sclerosis (MS), an 8-week clinical Pilates program produced no between-group differences in FoF or fall-risk indices, despite improvements in balance [30]. In contrast, a recent trial evaluating Dynamic Neuromuscular Stabilization demonstrated significant and sustained improvements in both fall rate and FoF, suggesting that targeted trunk and postural stabilization may influence both outcomes simultaneously in MS [31]. Overall, these findings suggest that simultaneous improvements in both falls and FoF are inconsistently reported across neurological conditions, although some targeted interventions may positively influence both outcomes. 

Several methodological factors likely contribute to the inconsistent intervention effects observed across trials. In addition, many included interventions were not specifically designed to simultaneously target both falls and FoF, and several control conditions incorporated educational or preventive elements, potentially reducing between-group contrasts and contributing to the modest pooled effects. Many studies employed relatively short intervention periods (4–12 weeks), which may be insufficient to induce the physiological adaptations necessary to significantly reduce the incidence of falls. Fall measurement methods also varied widely, ranging from retrospective self-report to prospective fall calendars, potentially leading to underreporting or inconsistent event capture. Similarly, FoF was assessed using different scales (e.g., FES, FES-I, single-item questions, and ABC), limiting comparability across studies. In addition, the content of usual care varied across studies and was not consistently reported. This variability may have influenced between-group comparisons and contributed to the heterogeneity observed across trials. Heterogeneity in participant characteristics, including baseline fall risk, comorbidities, and psychological profiles, further complicates interpretation. In particular, inconsistent reporting of comorbid conditions limited our ability to evaluate their influence on intervention effectiveness and may partly explain the heterogeneity observed across studies. In addition, the relatively small number of eligible studies likely reflects the narrow overlap between randomized controlled trials reporting both falls and FoF outcomes. These limitations underscore the need for more rigorous, longer-duration trials with standardized outcome measures and more transparent reporting of intervention fidelity.

A significant strength of this review is its focus on interventions that simultaneously measured both falls and FoF, allowing direct comparison across two outcomes that are commonly but incorrectly assumed to be linked. The inclusion of only RCTs strengthens causal inference. However, the small number of eligible studies, variability in outcome measures, and inconsistent follow-up durations limit the ability to draw firm conclusions about specific intervention types. Meta-analytic results should also be interpreted with caution due to heterogeneity in intervention components and participant populations.

## 5. Conclusions

Taken together, the findings of this systematic review and meta-analysis underscore that interventions targeting older adults rarely produce simultaneous improvements in both FoF and actual fall incidence. These findings may suggest that the psychological and physical determinants of fall risk operate through partly independent pathways and therefore require separate assessment and targeted intervention. Clinicians should routinely evaluate both domains and avoid assuming that gains in one will automatically translate to improvements in the other. Although multimodal and individually tailored programs may hold promise, current evidence remains limited, and further research is needed to develop and rigorously test integrated approaches capable of addressing the complex interplay between balance impairments, fear responses, and behavioral adaptations that influence fall risk in older adults.

## Figures and Tables

**Table 1:** Study and intervention characteristics.

First Author, Year, Country (*n*)	Study Design	Population	Mean Age	Key Eligibility Criteria	Dose	Intervention (Trainer)	Control Group	Fall and Fear of Falling Outcomes	Assessment Timepoints
Dorresteijn 2016 Netherlands [17] (*n* = 389)	RCT	Older adults ≥ 70	Intervention group: 78.38 (5.4) Control group: 78.25 (5.3)	Any concerns about falls	7 sessions (3 home visits + 4 calls)	A matter of balance (trained facilitators)	Usual care (no standard treatment for concerns about falls)	FoF: FES-I (Primary) Falls: Monthly fall calendar (Secondary)	1. Baseline 2. Month 4 [EOI] 3. Month 5 [FU] 4. Month 12 [FU]
Huang 2011 Taiwan [18] (*n* = 60)	RCT	Older adults ≥ 60	NA	Independent ambulation	CBT: 8 wks, 1x/wk, 60–90 min Tai Chi: 8 wks, 3x/wk, 60 min	CBT (trained facilitator) + Tai Chi (certified instructor)	Received fall-prevention brochure	FoF: FES (Primary) Falls: Self-reported falls (Primary)	1. Baseline 2. Month 2 [EOI] 3. Month 5 [FU]
Tennsledt 1998 USA [19] (*n* = 434)	RCT	Older adults ≥ 60	All participants: 77.8 (7.71)	FoF + activity restriction	4 weeks, 2x/wk, 2 h sessions	A matter of balance (trained facilitators)	Social contact: single 2 h group session	FoF: FES (Primary) Falls: Self-reported falls (Secondary)	1. Baseline 2. Week 6 [EOI] 3. Month 6 [FU] 4. Month 12 [FU]
Wetherell 2018 USA [20] (*n* = 42)	Pilot RCT	Older adults ≥ 65	Intervention group: 77.3 (7.0) Control group: 78.5 (7.8)	FoF (FES-I > 27)	8 weekly sessions	The ABLE intervention (physiotherapist)	Fall prevention education	FoF: FES-I(Primary) Falls: Falls recorded via paper calendars (Secondary)	1. Baseline 2. Month 2 [EOI] 3. Month 3 [FU] 4. Month 6 [FU]
Zijlstra 2009 Netherlands [21] (*n* = 540)	RCT	Older adults ≥ 70	Intervention group: 77.8 (4.6) Control group: 78.0 (5.0)	FoF + activity avoidance	8 weekly 2 h group sessions + booster at 6 wks	CBT-based group sessions (trained nurses)	Usual care (NA)	FoF: Single Question (Primary) Falls: Self-reported falls (Secondary)	1. Baseline 2. Month 2 [EOI] 3. Month 8 [FU] 4. Month 14 [FU]
Campbell 1999 New Zealand [22] (*n* = 103)	RCT	Older woman ≥ 80	Intervention group: 83.4 (2.7) Control group: 84.3 (3.3)	No physiotherapy	8 wks exercise + home exercises 3x/wk for 2 yrs	Home-based strength/balance training (physiotherapist)	Social visits	FoF: FES (Primary) Falls: Falls diaries returned monthly for 2 years	1. Baseline 2. First year [EOI] 3. Second year [FU]
Barnett 2003 Australia [23] (*n* = 163)	RCT	Older adults ≥ 65	Intervention group: 74.4 (4.9) Control group: 75.4 (6.0)	At risk of falling	1 h weekly groups x 1 yr (37 sessions) + home program	Supervised group-based balance & strength exercises (physiotherapist)	Practical fall-prevention strategies	FoF: Single question. (Primary) Falls: Prospective falls calendars completed monthly for 12 months (Secondary)	1. Baseline 2. Month 6 3. Month 12 [EOI]
Haines 2009 Australia [24] (*n* = 50)	Pilot RCT	Older adults ≥ 65 recently discharged from hospital	Intervention group: 77.3 (7.0) Control group: 78.5 (7.8)	Independent ambulation, discharged ≤ 2 wks	8 wks, video-based home exercise	Video-guided home exercises (no live supervision)	Usual care (did not receive exercise program materials, home visits, or telephone follow-up)	FoF: Activities-Specific Balance Confidence (Primary Outcome) Falls: Self-reported falls via monthly postcards + phone follow-up (Secondary)	1. Baseline 2. Month 2 [EOI] 3. Month 6 [FU]

FoF: Fear of Falling, EOI: End of Intervention, FU: Follow Up, FES-I: Falls Efficacy Scale—International, FES: Falls Efficacy Scale, NA: Not available.

**Table 2:** Items of the PEDro risk of bias assessment.

Author (Year)	Eligibility	Random Allocation	Concealed Allocation	Baseline Comparability	Blinding Subjects	Blinding Therapist	Blinding Assessors	Adequate Follow-Up	Intention-to-Treat Analysis	Between-Group Statistical Comparison	Point Measures and Measures of Variability	Total Score (0 to 11)
Dorresteijn (2016) [17]	Y	Y	Y	Y	N	N	Y	N	Y	Y	Y	8
Huang (2011) [18]	Y	Y	Y	Y	N	N	Y	Y	Y	Y	Y	9
Tennstedt (1998) [19]	Y	N	N	N	N	N	Y	Y	Y	Y	Y	7
Wetherell (2018) [20]	Y	Y	Y	Y	N	N	Y	Y	N	Y	Y	9
Zijlstra (2009) [21]	Y	Y	Y	Y	N	N	Y	N	Y	Y	Y	8
Campbell (1999) [22]	Y	Y	N	Y	N	N	Y	N	Y	Y	Y	7
Barnett (2003) [23]	Y	Y	Y	Y	N	N	Y	N	Y	Y	Y	8
Haines (2009) [24]	Y	Y	Y	Y	N	N	Y	N	N	Y	Y	7
Logghe (2009) [25]	Y	Y	Y	Y	N	N	Y	N	Y	Y	Y	8
Sihvonen (2004) [26]	Y	Y	Y	Y	N	N	N	N	N	Y	Y	6

**Table 3:** Detailed findings of the included studies.

	Outcome	Baseline (A)	EOI (B)	Follow-Up (C)	Statistical Significance
Dorresteijn, 2016 [17]	FoF (Intervention)	35.70 (10.4)	-	31.73 (10.4) (5 mo.) 31.98 (10.9) (12 mo.)	A-C: *p* < 0.001; Effect size (d) = 0.34 A-C: *p* < 0.001; Effect size (d) = 0.35
	FoF (Control)	35.47 (9.4)	-	35.30 (10.4) (5 mo.) 35.86 (11.1) (12 mo.)	-
	Falls (Intervention)	128/194 fallers (66.0%)	-	94/166 fallers (56.6%, 12 mo.)	A-C (Intervention & Control): OR: 0.79; *p* = 0.292
	Falls (Control)	111/194 fallers (57.2%)	-	106/180 fallers (58.9%)	
Huang, 2011 [18]	FoF (Intervention)	CB: 88.81 (17.40) CB + Thai Chi: 94.26 (16.71)	CB: 90.1 (16.9) CB + Thai Chi: 96.7 (15.0)	CB: 90.88 (15.72) CB + Thai Chi: 99.14 (11.66)	A-C (CB + Thai Chi &CB &Control): *p* = 0.061
	FoF (Control)	90.4 (16.8)	88.7 (20.1)	88.40 (18.71)	
	Falls (Intervention)	CB: 8/62 fallers (%12.9) CB + Thai Chi: 8/62 fallers (8.1%)	-	CB: 8/60 fallers (%13.3) CB + Thai Chi: 3/56 fallers (5.4%)	A (CB + Thai Chi & CB & Control): *p* = 0.62
	Falls (Control)	8/62 fallers (%12.9)	-	8/60 fallers (%13.3)	C (CB + Thai Chi & CB & Control): *p* = 0.28
Tennsledt, 1998 [19]	FoF (Intervention)	-	Change from baseline: 0.15	Change from baseline (mo. 12): 0.09	A-B change: *p* < 0.01 A-C change: *p* < 0.01
	FoF (Control)	-	Change from baseline: −0.04	Change from baseline (mo. 12): −0.12	A-B change: *p* > 0.05 A-C change: *p* > 0.05
	Falls (Intervention)	-	Change from baseline: −0.25	Change from baseline: −0.27	A-B change: *p* > 0.05 A-C change: *p* > 0.05
	Falls (Control)	-	Change from baseline: −0.23	Change from baseline: −0.16	A-B change: *p* > 0.05 A-C change: *p* > 0.05
Wetherell, 2018 [20]	FoF (Intervention)	40.7 (7.3)	30 (2.5)	31 (3.0) (month 6)	A (Intervention & Control): *p* = 0.44 B (Intervention & Control): *p* = 0.02 C (Intervention & Control): *p* = 0.46
	FoF (Control)	39.0 (6.8)	35 (2.5)	34 (3.0) (month 6)	
	Falls (Intervention)	15/21 fallers (81.4%)	4/21 fallers (19.0%)	9/19 fallers (47.3%)	A (Intervention & Control): *p* = 0.19 B (Intervention & Control): *p* = 0.83 C (Intervention & Control): *p* = 0.29
	Falls (Control)	12/21 fallers (57.2%)	3/21 fallers (14.3%)	4/21 fallers (19.0%)	
Zijlstra, 2009 [21]	FoF (Intervention)	112/280 has FoF (40.0%)	37/280 has FoF (16.3%)	48/280 has FoF (24.5%)	A (Intervention & Control): OR: 0.11; *p* < 0.001
	FoF (Control)	116/260 has FoF (44.6%)	101/260 has FoF (43.3%)	86/260 has FoF (41.7%)	
	Falls (Intervention)	-	46/226 fallers (20.4%)	91/118 fallers (48.4%)	A to B (Intervention & Control): OR: 0.96; *p* = 0.92 A to C (Intervention & Control): OR: 0.50; *p* = 0.08
	Falls (Control)	-	50/236 fallers (21.2%)	117/203 fallers (57.6%)	
Campbell, 1999 [22]	FoF (Intervention)	91.1 (11.0)	93.3 (9.4)	-	A (Intervention & Control): *p* > 0.05 B (Intervention & Control): *p* = 0.03
	FoF (Control)	90.6 (10.6)	86.8 (15.1)	-	
	Falls (Intervention)	-	88 falls (overall rate: 0.81)	138 falls (overall rate: 0.83)	B: Relative Hazard Ratio: 0.68 C: Relative Hazard Ratio: 0.69
	Falls (Control)	-	152 falls (overall rate: 1.34)	220 falls (overall rate: 1.19)	B: Relative Hazard Ratio: 1.0 C: Relative Hazard Ratio: 1.0
Barnett, 2003 [23]	FoF (Intervention)	14/83 has FoF (16.9%)	5/67 has FoF (7.5%)	-	C (Intervention & Control): Adjusted for baseline, *p* > 0.05
	FoF (Control)	9/80 has FoF (11.3%)	6/70 has FoF (8.6%)	-	
	Falls (Intervention)	36/83 fallers (43.4%)	-	27/76 fallers (35.5%)	C (Intervention & Control): Relative Ratio: 0.71 (0.49–1.04)
	Falls (Control)	33/80 fallers (41.3%)	-	37/74 fallers (50.0%)	
Haines, 2009 [24]	FoF (Intervention)	5.3 (2.1)	-	5.3 (2.0)	A (Intervention & Control): *p* = 0.69 C (Intervention & Control): β: −0.30, *p* = 0.61
	FoF (Control)	5.5 (1.9)	-	5.6 (2.0)	
	Falls (Intervention)	28 falls	-	21 falls [11 fallers (57.89%)]	C (Intervention & Control): Incidence Rate Ratio: 0.72, *p* = 0.41 OR: 0.96, *p* = 0.95
	Falls (Control)	74 falls	-	51 falls [20 fallers (58.82%)]	
Logghe, 2009 [25]	FoF (Intervention)	6.0 (5.0)	4.9 (4.4)	5.2 (4.8)	A (Intervention & Control): *p* = 0.47 B (Intervention & Control): *p* = 0.38 C (Intervention & Control): *p* = 1.00
	FoF (Control)	5.7 (5.0)	5.8 (5.3)	5.7 (4.7)	
	Falls (Intervention)	88/138 fallers (63.8%)	-	95 falls in previous fallers	C (Intervention & Control): Hazard ratio: 1.38, *p* > 0.05
	Falls (Control)	79/131 fallers (60.3%)	-	59 falls in previous fallers	
Sihvonen, 2004 [26]	FoF (Intervention)	13/20 has FoF (65.0%)	8/20 has FoF (40.0%)	12/20 has FoF (63.0%)	A to B: *p* = 0.02
	FoF (Control)	5/7 has FoF (71.0%)	6/7 has FoF (86.0%)	6/7 has FoF (86.0%)	
	Falls (Intervention)	7/20 fallers (35.0%)	-	11/20 fallers (55.0%)	A (Intervention & Control): *p* = 1.0 A to C (Intervention & Control): Risk Ratio: 0.398; *p* = 0.029
	Falls (Control)	2/7 fallers (29.0%)	-	5/7 fallers (71.0%)	

## Data Availability

The data that support the findings of this systematic review are available from Supplementary Materials/the corresponding author upon reasonable request.

## Supplementary Materials

The following supporting information can be downloaded at: https://www.mdpi.com/article/10.3390/geriatrics11030072/s1, Table S1: PRISMA 2020 Checklist.
